# An Overview of Pharmacotherapy in the Management of Children with Autism Spectrum Disorder at a Public Hospital in KwaZulu-Natal

**DOI:** 10.1007/s10578-023-01514-z

**Published:** 2023-03-22

**Authors:** Jennal Maniram, Frasia Oosthuizen, Saira B.S. Karrim

**Affiliations:** School of Health Sciences, College of Health Sciences, University of KwaZulu-Natal, Durban, South Africa

**Keywords:** Pharmacotherapy, Therapeutic outcome, Core symptom, Comorbidity, Autism

## Abstract

This study presents an overview of prescribing patterns and provides insight into the current management practice for the core symptoms and comorbidities of ASD in children. A quantitative retrospective study was conducted at a public hospital in KwaZulu-Natal, South Africa by reviewing patient files of children diagnosed with ASD and meeting the inclusion criteria for the study. A descriptive analysis of data was done to identify treatment trends and patient therapeutic outcomes. A total of 181 children met the inclusion criteria of the study. Risperidone was the most frequently prescribed drug (88%) for the management of comorbidities and/or core symptoms of ASD. Drugs prescribed to manage ASD comorbidities included methylphenidate, melatonin, sodium valproate, risperidone, oxybutynin, carbamazepine, and others. Except for risperidone, there were no additional drugs that targeted the core symptoms of ASD. Non-pharmacological therapies were often used collaboratively with medication to manage ASD symptoms. In 41% of patients, there were improvements in their symptoms.

## Introduction

Autism Spectrum Disorder (ASD) is a developmental disorder that involves impairment of social and communication abilities, restricted interests or repetitive behaviours, and challenges with sensory processing. The term “spectrum” reflects the fact that symptoms vary between individuals, ranging in type and severity [1].

To meet diagnostic criteria for ASD, according to the Diagnostic and Statistical Manual of Mental Disorders, 5th Edition (DSM-5), a child must have persistent deficits in each of three areas of social communication and interaction plus at least two of four types of restricted, repetitive behaviours [2]. Proposed DSM-5 ASD criteria include three severity classifications: Level 1 (“Requiring support”), Level 2 (“Requiring substantial support”), and Level 3 (“Requiring very substantial support”) [3]. Prescribers often describe or identify level 1 as mild ASD, level 2 as moderate ASD, and level 3 as the most severe form of ASD [4, 5].

Medical comorbidities are more common in children with ASD than in the general population and can include epilepsy, macrocephaly, cerebral palsy, migraine/headaches, congenital abnormalities of the nervous system, gastrointestinal disorders, sleep disorders, allergic disorders, and persistent neuroinflammation [6].

According to the World Health Organisation (WHO), approximately 1 in 100 children have ASD; however, these figures could be substantially higher based on results from additional well-controlled studies and the absence of ASD statistics in various low and middle-income countries [7]. In South Africa, accurate local statistics for ASD are not available but it has been estimated that between 1% and 2% of the population may be affected by ASD [8].

The Food and Drug Administration (FDA) has approved the use of antipsychotic drugs, risperidone, and aripiprazole, for treating irritability associated with ASD in children. Other drugs are often used to improve symptoms of ASD, but are not approved by the FDA for this specific purpose; these include selective serotonin reuptake inhibitors, tricyclic antidepressants, stimulants, anticonvulsants, antipsychotics, and anti-anxiety medication [9]. There are no approved medications for ASD core symptoms; however, given the significant clinical need, individuals with ASD are prescribed medication off-label for core or associated conditions, sometimes based on limited evidence for effectiveness [10].

Although some studies have evaluated and reviewed the pharmacological management of ASD [11, 12], there is limited data available on the pharmacological management of ASD in South Africa and Africa. The only known study in South Africa that assessed the prevalence and patterns of medication use in children with ASD was conducted almost a decade ago and utilised a survey questionnaire [13].

This study aims to review pharmacological treatment options used in the management of ASD in children at a public hospital in KwaZulu-Natal by identifying pharmacological therapeutic agents prescribed and determining their role and impact on treatment outcomes.

## Methodology

### Study Design and Setting

A retrospective descriptive study was carried out to determine the pharmacological management of children diagnosed with ASD. The study setting was a public sector referral hospital located in the KwaZulu-Natal province of South Africa. This hospital was initially built in 1928 as the first facility in Africa dedicated exclusively to the treatment of children. A neuro-developmental assessment centre is located at the hospital and comprises specialised paediatric neurologists and a multidisciplinary team that manages children diagnosed with ASD and other neurological disorders.

### Study Target Population and Sampling Strategy

#### Inclusion Criteria

Patient files that met the following criteria were included in the study:

- Male and female individuals diagnosed with ASD (at all levels of severity).
- Children and adolescents between the age group of 2 and 17 years of age.
- ASD individuals with hospital visits between 01 and 2019 to 31st January 2022.
- ASD individuals with any type of medical comorbidity.

#### Exclusion Criteria

- Neurology patients who were not on the autism spectrum according to the DSM-5 criteria [14].
- ASD patients who have not been prescribed any type of medication to manage the core symptoms or comorbidities.
- Individuals over the age of 17.

A total of 181 patients met the inclusion criteria and this sample size was deemed large enough to allow a detailed analysis of the pharmacological management of ASD.

### Data Collection

Patient medical files were the primary data source. A data extraction template was used to enter all required information for the retrospective study. The age, gender, and information related to name, dose, and frequency of medication/s prescribed were recorded. The prescriber’s notes on the patient’s progress and therapeutic outcomes were also obtained from the patient’s medical file. An improvement or decline in the patient’s condition was noted as an indicator of the therapeutic outcome. ASD-related comorbidities that warranted pharmacological treatment were determined from the patient file and were also identified by the class of drugs prescribed to treat a particular condition. Additionally, non-pharmacological interventions in the form of rehabilitation (speech, occupational, or behaviour therapy) were obtained from the patient’s file and recorded on the data template.

### Data Analysis

Data were analysed using descriptive statistics and presented in the form of frequency tables and bar and column graphs. T-tests were conducted to determine the probability (p-value) of results when required. To eliminate bias, data were collected utilising a pre-designed tool and the extraction of data was performed solely by the corresponding author of the study, with no assistance from medical staff who prescribed or dispensed the medication. The study eliminated selection bias by including all available patient files, dating from 01 to 2019 to 31 January 2022, meeting the inclusion criteria of the study.

### Data Confidentiality and Ethical Approval

To maintain confidentiality, no information that could identify patients was extracted during the data collection process. Since this study obtained retrospective information from patient files, with no direct contact with patients, informed consent was not required. Ethical approval and consent for access to the patient files were obtained from the Biomedical Research and Ethics Committee (BREC) of the University of KwaZulu-Natal (UKZN), the provincial office of the KwaZulu-Natal Department of Health (KZN-DOH) and the eThekwini district office of KZN-DOH.

## Results

### Demographic and General Characteristics of the Study

The final study sample consisted of 181 patient files and the demographic data is presented in Table 1. In this study, the ASD gender ratio was found to be approximately 3:1 with males consisting of 77% of the study sample compared to 23% of females. The ages of children diagnosed with ASD were between 2 and 13 years old with the majority of children presenting to the hospital between 4 and 6 years of age (61%).

**Table 1:** The Demographic Characteristics of the Sample (n = 181)

Characteristics of Children		Number	%
Sex	Male	139	77
	Female	42	23
Age Groups	2–3 years	24	13
	4–6 years	111	61
	7–9 years	40	22
	10–13 years	24	13
ASD Level of Severity	Mild (Level 1)	32	18
	Moderate (Level 2)	111	61
	Severe (Level 3)	38	21

### Medication Prescribed for Children Diagnosed with ASD

**Table 2:** List and Characteristics of Medication Prescribed

Name of Medication	Medication Class	Dosing Range	Therapeutic Indication in Study	Frequency of Drug Choice
Risperidone	Antipsychotic	0.1 mg – 2 mg	Irritability, aggression, disruptive behaviour, sleep dysregulation, hyperactivity symptoms, and core symptoms of ASD	n = 159 (88%)
Aripiprazole	Antipsychotic	2,5 mg – 5 mg	Indicated for use in the absence of response to risperidone.	n = 1 (0, 6%)
Citalopram	Antidepressant	10 mg	Anxiety, self-injurious and obsessive-compulsive behaviours	n = 2 (1, 1%)
Sodium Valproate	Anticonvulsant	120 mg – 400 mg	Epilepsy	n = 29 (16%)
Carbamazepine	Anticonvulsant	40 mg – 200 mg	Epilepsy, sleep dysregulation, or as a mood stabiliser	n = 17 (9%)
Lamotrigine	Anticonvulsant	5 mg – 75 mg	Epilepsy	n = 8 (4, 4%)
Topiramate	Anticonvulsant	6,25 mg – 75 mg	Epilepsy	n = 6 (3, 3%)
Levetiracetam	Anticonvulsant	125 mg – 500 mg	Epilepsy	n = 3 (1, 7%)
Clonazepam	Benzodiazepine	0,1 mg – 0,6 mg	Epilepsy	n = 4 (2, 2%)
Methylphenidate	Stimulant	2,5 mg – 20 mg	Attention Deficit Hyperactivity Disorder (ADHD), hyperactive symptoms, reduced attention span, and concentration problems	n = 58 (32%)
Clonidine	Alpha Agonist	0,25 mg	Sleep dysregulation or hyperactivity	n = 7 (3, 9%)
Melatonin	Sedative	3 mg	Sleep dysregulation	n = 5 (2, 8%)
Oxybutynin	Anticholinergic	5 mg – 10 mg	Enuresis	n = 4 (2, 2%)
Cetirizine	Antihistamine	5 mg – 10 mg	Allergic rhinitis	n = 4 (2, 2%)
Fluticasone nasal spray	Steroid	unknown	Allergic rhinitis	n = 3 (1, 7%)
Lactulose	Laxative	5ml – 10ml	Constipation	n = 8 (4, 4%)
Liquid Paraffin	Laxative	5ml	Constipation	n = 1 (0, 6%)
Folic Acid	Vitamin B9	2,5 mg – 5 mg	As a dietary supplement when sodium valproate was prescribed.	n = 7 (3, 9%)

Table 2 provides a detailed list of all medications prescribed to children with ASD and includes the drug class, dosing range, therapeutic indication, and frequency of use. Risperidone was the most frequently prescribed drug (88%), followed by methylphenidate (32%) and sodium valproate (16%). Examples of common medication classes prescribed were antipsychotics (risperidone), anticonvulsants (lamotrigine, sodium valproate, topiramate, and levetiracetam), stimulants (methylphenidate), and mood stabilisers (carbamazepine).

### The Pharmacological Management of ASD-related Comorbidities

The comorbidity mostly present in children diagnosed with ASD was hyperactivity or ADHD (57%). The most frequent pharmacological agents prescribed to manage the associated ADHD included risperidone (51%), methylphenidate (58%), and clonidine (4%). Irritability, aggression, or disruptive behaviour, appearing in 27% of study subjects, was managed with risperidone.

Sleep dysregulation also appeared frequently (22%) in children diagnosed with ASD and the therapeutic agents prescribed to manage this condition were melatonin (13%), clonidine (8%), risperidone (73%), or carbamazepine (8%), administered at night.

Epilepsy occurred in 16% of study subjects and was mostly managed with sodium valproate (97%) as monotherapy or in combination with other anticonvulsants including lamotrigine (27%), topiramate (20%), carbamazepine (13%), clonazepam (13%), or levetiracetam (10%).

Constipation was reported in 4% of children in the study and was managed by non-pharmacological agents in the form of lactulose (89%) or liquid paraffin (11%).

**Fig. 1:**
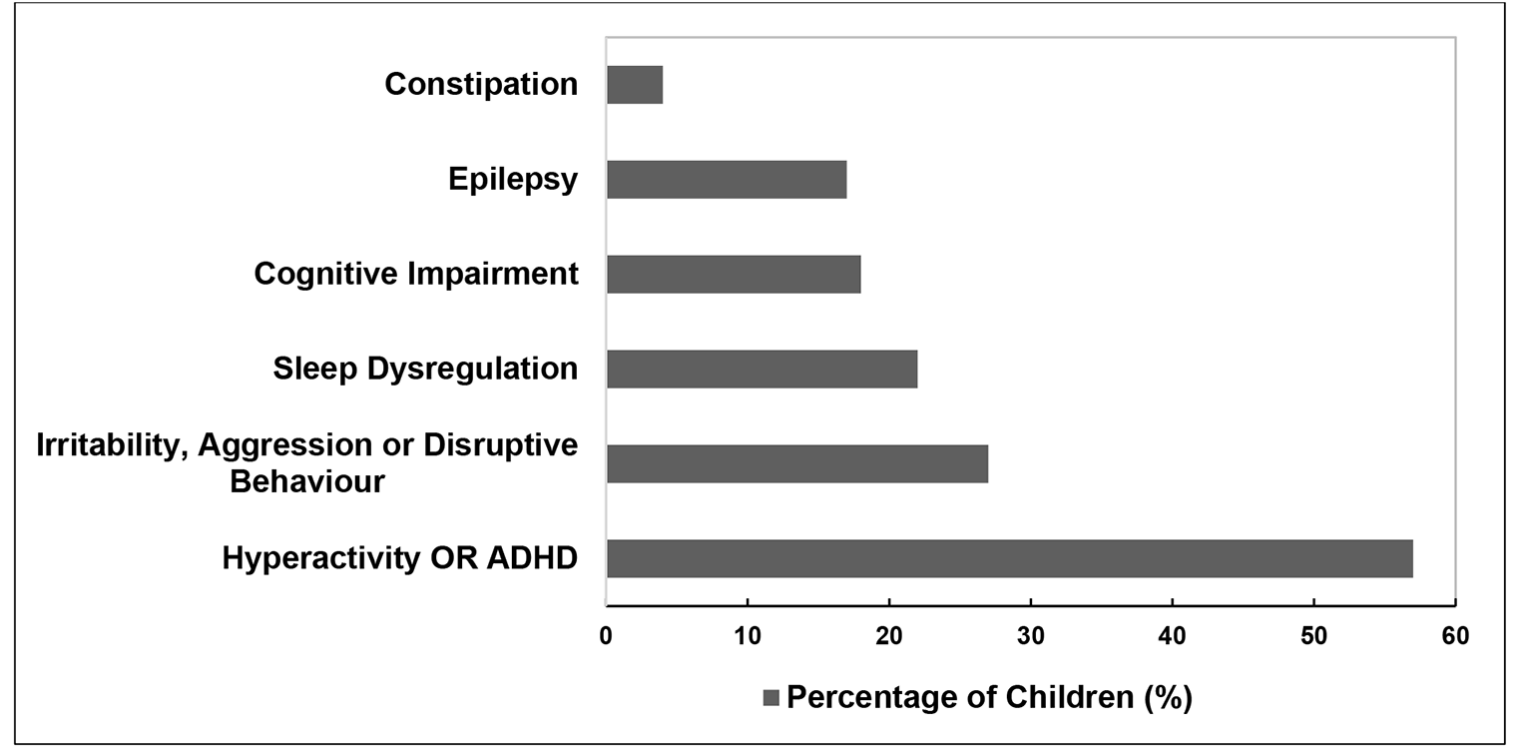
Common ASD comorbidities, presented by children in the study

Other ASD comorbidities that required pharmacological intervention were allergic rhinitis (treated with cetirizine and/or fluticasone nasal spray) and nocturnal enuresis (treated with oxybutynin). Citalopram, an antidepressant, was prescribed for two children that displayed self-injurious behaviour. Figure 1 provides an overview of the most frequent comorbidities experienced by children with ASD.

### The Prescribing of Psychotropic Drug Combinations

In this study, examples of psychotropic drugs prescribed included risperidone, aripiprazole, citalopram, clonazepam, methylphenidate, melatonin, and carbamazepine.

**Fig. 2:**
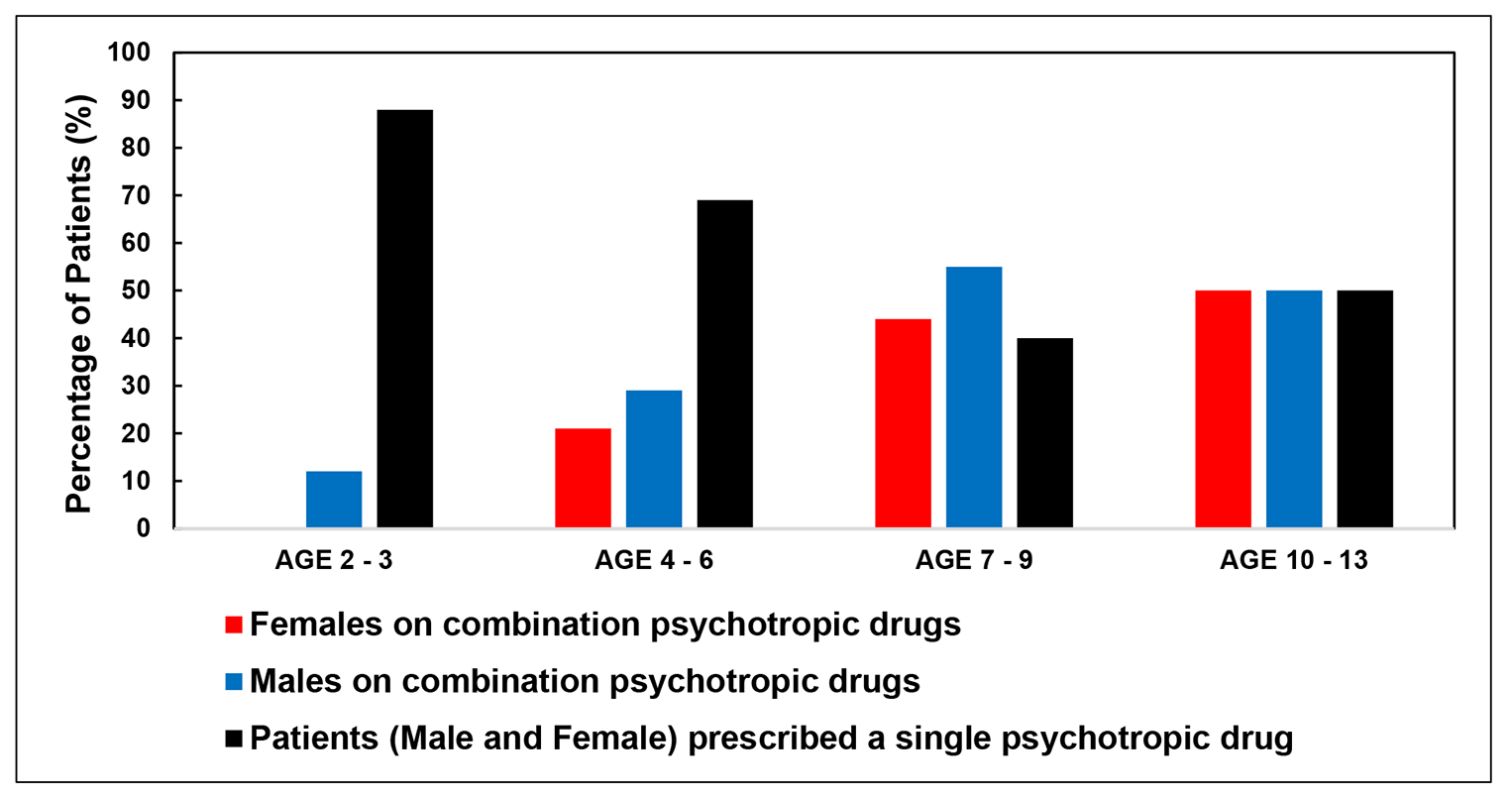
Relationship between age and gender and the percentage of children prescribed psychotropic drugs

Figure 2 illustrates the relationship between age, gender, and the prescribing of two or more psychotropic drugs. Most children in the 2–3-year-old age group were prescribed risperidone as monotherapy to control ASD symptoms; however, there were 2 male children in this age group that was prescribed methylphenidate (n = 1) or melatonin (n = 1) in addition to risperidone.

In the 4–6-year age group, combination prescribing was very similar when comparing males (29%) to females (21%). In the 7–9-year age group, significantly more males were on two or more psychotropic drugs when compared to other age groups (p-value = 0.03). In older children (ages 10–13), equal numbers of males and females were prescribed two or more psychotropic drugs. Additionally, Fig. 2 also illustrates the percentage of children prescribed a single psychotropic drug to manage ASD symptoms. This percentage decreased as the age of the child increased as shown with the highest percentage seen in the 2 -3-year-old age group and the lowest in the 7–9-year-old age group. This demonstrates the need for combination psychotropic drugs as the age of the child increased. The children in the 10–13-year-old age group however did not follow this trend since 50% of children were prescribed methylphenidate as a single agent, mainly for challenges with concentration at school. This could be an indication of psychiatric comorbidities that warrant the use of combination psychotropic drugs, possibly peaking in children between the ages of seven and nine however additional studies are required to confirm this.

### Therapeutic Outcomes

The therapeutic outcomes for ASD management were obtained from prescriber progress notes in the patient files. In 41% (n = 74) of patients, there was a positive response to treatment due to an overall improvement in an ASD comorbidity and/or core symptoms from previous visits. In 20% of all patients, the core symptoms of ASD improved (defined by improvements in social communication, improved shared interest, and a decrease in repetitive behaviours).

Of the 41% (n = 74) of patients with improved treatment outcomes, 85% (n = 63) were prescribed risperidone. Other drugs associated with improved therapeutic outcomes included methylphenidate (34%), sodium valproate (19%), and carbamazepine (9%). These drugs were either prescribed as monotherapy or in a combination with other drugs and/or non - pharmacological therapies.

Examples of qualitative comments from the prescriber related to improved therapeutic outcomes were; “hyperactivity and meltdowns have improved”, “behaviour has markedly improved on risperidone, he is no longer aggressive, now sleeps well and can also make 3-word sentences”, “from the initial visit his aggressive behaviour has improved”, “he is now toilet trained and says occasional words”, “he has improved in his communication and stereotypic behaviour”, “sleep and hyperactivity have improved with risperidone”, “she has improved in communication, talks sentences, and methylphenidate improved her behaviour” and “his shared interest has improved from the last visit”.

In 31,5% (n = 57) of patients, there was a decline in their condition, described as worsening of symptoms, the need to increase doses of pharmacotherapy, and/or add on additional drug treatment/s and/or non-pharmacological therapies (speech, occupational, and behaviour therapy). Few patients (5%), displayed a negative response to methylphenidate as it either made the child’s behaviour worse, resulted in a poor appetite, caused a deterioration in behaviour once the dose of the drug wore off, or displayed minimal effectiveness leading the prescriber to discontinue the drug. Additionally, there were a few patients (2, 8%), who were prescribed risperidone which either resulted in significant obesity, vomiting or caused a worsening of behaviour leading the prescriber to initiate alternative pharmacotherapy.

In 21, 5% (n = 39) of patients, there were no notes related to treatment outcomes since their patient file only contained notes on an initial visit. These patients were either referred to their base hospital or would return for a review during the year.

In the balance of patients (n = 11), there were mixed comments related to therapeutic outcomes as the prescriber indicated that some symptoms improved yet others persisted and, for some patients, it was suggested that intensive non-pharmacological therapies be continued or commenced despite improvements in certain ASD comorbidities.

## Discussion

This study provides an overview of the pharmacological management of ASD in children and adolescents through a retrospective review of patient medical files at a public hospital in KwaZulu-Natal, South Africa. A comprehensive list and description of pharmacological agents prescribed were analysed and reviewed.

While a number of pharmacological agents were found to be used in children with ASD, risperidone was the drug used most often. Risperidone is an FDA-approved agent for use in targeting the irritability and aggression associated with ASD in children [9]. In this study, risperidone was prescribed to assist in the management of several comorbidities (irritability, aggression, disruptive behaviour, hyperactivity, and insomnia) as well as the core symptoms of ASD. Though its precise mechanism of action is not fully understood, the current focus is on the ability of risperidone to inhibit the D2 dopaminergic receptors and 5-HT2A serotonergic receptors in the brain [15]. These dopamine and serotonin receptor antagonisms achieved by risperidone are believed to be responsible for the beneficial effects on ASD. The effects of risperidone on aggression, irritability stereotypes, tantrums, and restlessness can be attributed to dopamine antagonism. The effects on communication skills, restricted activity patterns and the ability to respond emotionally and socially can be considered a result of serotonin antagonism [16].

Although both risperidone and aripiprazole are FDA-approved agents for use in ASD [17], aripiprazole was only prescribed after consultation with a psychiatrist for a single patient with severe ASD who did not respond to risperidone. The limited use of aripiprazole would be an indication of adherence to standard treatment guidelines in South Africa, which lists risperidone as the only drug for the management of irritability, aggression, or self-injurious behaviour in children diagnosed with ASD [18]. The switch from risperidone to aripiprazole in this study is in line with an international prospective study which concluded that aripiprazole might be generally well tolerated and may constitute an alternative treatment for individuals with ASD who experience poor efficacy or tolerability issues with risperidone treatment [19].

In addition to risperidone, methylphenidate was also frequently prescribed to children in the study. This could be due to 57% of children presenting with ADHD or hyperactivity symptoms. Methylphenidate is pharmacologically classified as a stimulant and is widely used as the first-line treatment in children with ADHD. The mechanism of action of methylphenidate is related to the release of dopamine and norepinephrine in the central nervous system [20]. Various international studies [21–23] also support the use of methylphenidate to manage hyperactivity and ADHD symptoms in children diagnosed with ASD.

Sodium valproate was also a frequently prescribed drug (16%) and indicated for the management of epilepsy or seizures in this study. A variety of other drugs were also prescribed to manage epilepsy symptoms and included lamotrigine, clonazepam, levetiracetam, and topiramate. Epilepsy is reported to co-occur in individuals with ASD and studies across the world have found prevalence estimates ranging from 4 to 38% [24]. Additionally, several lines of evidence point to valproate, lamotrigine, and levetiracetam as the most effective and tolerable anti-epileptic drugs for individuals with ASD [25]. Although there are limited studies on the pharmacological management of epilepsy in children diagnosed with ASD, there is no evidence that seizures in children with ASD respond any differently to anti-epileptic drugs than seizures in children without ASD [26].

Cognitive impairment appeared in 16% of study subjects with ASD; however, it was unclear if any medication was prescribed to directly influence the management of this impairment. Additionally, neurodevelopmental regression appeared in 4% of children in this study. This condition is characterised by an initial normal social, emotional, and language development followed by loss of speech and social skills for no discernible reason [27]. All children that presented with this condition were prescribed risperidone, either to treat additional comorbidities that the child presented with, and/or positively influence the symptoms of this condition. An interesting yet distressing observation was that a large number of children (48%) presented with two or more medical comorbidities simultaneously. According to the DSM-5, 70% of the time a diagnosis of ASD is accompanied by an additional comorbidity, and 40% of the time by two or more additional comorbidities of diagnosis [28]. Additionally, an international study conducted to investigate the possibility of predictive patterns of ASD comorbidities in children, found there was an increased risk for seizures and sleep problems to co-occur with gastric disturbances, and behavioural impairments were also more severe as the number of co-occurring medical symptoms increased [29].

Common psychiatric comorbidities presented by children in this study included ADHD, aggression, disruptive behaviour, irritability, sleep dysregulation, anxiety, self-injurious behaviour, cognitive impairment, and neuro regression. Psychotropic medications are frequently used to target psychiatric symptoms in children with developmental conditions, despite limited evidence for their efficacy therefore clinicians should always use these drugs with caution, carefully weighing risks and benefits, and as a part of a comprehensive personalized approach [30]. In this study, the likelihood of psychotropic drug combinations among children with ASD was highest among the 7–9-year-old age group (p = 0.03) and it was also evident that males across all age groups required a combination of psychotropic drugs more often than females. This is in line with a recent study that concluded that during early childhood, girls with ASD tend to show a greater reduction and less rise in their ASD symptom severity than boys with ASD [31].

The core symptoms of ASD recorded in this study included restricted, repetitive behaviours and social communication challenges. Despite the limited treatment options for ASD core symptoms, 20% of children displayed significant improvements in social communication impairments and/or restricted repetitive behaviours and the majority (83%) of them were prescribed risperidone either to target the core symptoms of ASD and/or manage a comorbidity. Various international studies also support the use of risperidone for the management of the core symptoms either as monotherapy [32] or with the addition of other therapeutic agents [33, 34]. Targeting the core symptoms of ASD is of vital importance as these symptoms define ASD. A suggestion would be for prescribers to research current literature available regarding additional effective pharmacological management options for ASD core symptoms. As an example, recent clinical trials that utilise therapeutic agents like bumetanide [35], intranasal oxytocin [36], guanfacine [37], intranasal vasopressin [38], vitamin D [39], and omega fatty acids [40] have all demonstrated promising results on the core symptoms of ASD. It’s important to note that these therapeutic agents are still under study and evaluation and their usage to manage the core symptoms of ASD also depends on the availability of a drug in a particular country.

It is important to highlight the use of non – pharmacological therapies in conjunction with pharmacotherapy in the management of ASD in children. Non-pharmaceutical therapy can effectively relieve the core symptoms of ASD, has fewer side effects than drugs, and is easily accepted by patients [41]. In this study, 44% of patients were receiving speech and/or occupational therapy and in 23% of patients, these types of therapies were recommended by the prescriber. Currently, no medication can cure ASD or manage all of its symptoms, however, research shows that medication is most effective when used in combination with behavioural therapies [42].

Previous international studies on the pharmacological management of ASD focus mainly on psychotropic drug use [43–45] while other studies involve reviews on drug treatment for a single type of ASD comorbidity [46–48] or an additional study focused on the prevalence of co-occurring conditions and medication use in the management of comorbidities among individuals with ASD [49]. This study provides a review of the pharmacological management of comorbidities and core symptoms of ASD, therapeutic outcomes, and psychotropic drug usage in children diagnosed with ASD. Additionally, the results from the study were compared to various international trends. Interestingly, this is also the first known type of study to be conducted in a clinical setting in South Africa. This is taking into consideration the study in the Western Cape related to the pharmacological management of ASD, conducted almost a decade ago in a school with a sample size of 65 pupils [13]. As a recommendation, additional studies could be conducted in other provinces and could also include the private sector to obtain a more generalised summary of the role of pharmacotherapy in the management of ASD in South Africa.

### Limitations

Study results are from a single hospital based in one district of the province of KwaZuIu- Natal, however, this was a referral hospital and included patients from different locations throughout the province. The study was conducted in a public hospital setting therefore the therapeutic agents prescribed may differ from a private hospital setting as public hospitals in South Africa are required to adhere to standard treatment guidelines [18] and essential drug lists [18] when prescribing medication. The study included a review of patient files with initial hospital visits from January 2019 to January 2022 only, therefore it does not reflect the total number of ASD patients that have visited the hospital. Additionally, the hospital utilises a manual filing system and this presented the challenging task of searching through the medical records of every patient that visited the hospital to locate patients that met the study inclusion criteria. Furthermore, medical records were filed according to the child’s date of birth, and ASD patients were not filed separately from patients with other types of neurological disorders. Due to these challenges with locating patients, it is difficult to interpret the 181 patients included in the study as an exact numerical representation of patients that met the inclusion criteria for this study. Additional limitations of this study that require attention include the relatively young age of the sample and the majority of patients presenting with a moderate form of ASD with regards to severity. This further elaborates the point that this sample may not be fully representative of the province or country of South Africa.

### Summary

The pharmacological management of autism remains a challenge due to limited effective treatment options, FDA approval of only two drugs, and the absence of drugs that can cure the core symptoms. In South Africa, little is known about the role of pharmacotherapy in the management of children diagnosed with ASD. This study provided insight into the current pharmacological management of ASD in children at a KwaZulu-Natal hospital. Risperidone was the most frequently prescribed drug (88%) for the management of comorbidities and/or core symptoms of ASD. This study also provided sufficient information to gain clarity on the role of pharmacotherapy in the management of ASD. Based on the results of this study, various drugs (for example risperidone, methylphenidate, sodium valproate, oxybutynin, melatonin, and others) play a prominent role in managing and improving common ASD-related comorbidities like irritability, aggression, sleep dysregulation, epilepsy, ADHD and nocturnal enuresis. There was a challenge regarding limited treatment options for ASD core symptoms however this was expected considering there are still no approved therapeutic agents that can cure these symptoms [10]. Although a few patients that were prescribed risperidone experienced improvements in the core symptoms of ASD, the direct role of pharmacotherapy in the management of ASD core symptoms remains questionable. The frequent prescribing of 2 or more psychotropic drugs to manage psychiatric comorbidities (for example aggression, self-injurious and disruptive behaviour, irritability, and ADHD) in this study is also a noteworthy area of concern as these drugs come with high side effect profiles and their use should be limited to when the benefit outweighs the risk to the child. From the results of this study, 41% of patients achieved a positive prognosis and this number could increase substantially depending on the therapeutic outcomes for children with initial visits. As the availability and use of effective drugs (including vitamin, mineral, and dietary supplements) for the management of ASD core symptoms increases and with the collaborative use of intensive non-pharmacological interventions, it is safe to conclude that substantially greater therapeutic outcomes could be achieved for children diagnosed with ASD in South Africa.

## Data Availability

The data that support the findings of this study are available from the corresponding author upon reasonable request.
